# Learning self-care skills after spinal cord injury: a qualitative study

**DOI:** 10.1186/s40359-021-00659-7

**Published:** 2021-10-09

**Authors:** Tijn van Diemen, Ilse J. W. van Nes, Charlotte C. M. van Laake-Geelen, Dorine Spijkerman, SELF-SCI Group, Jan H. B. Geertzen, Marcel W. M. Post

**Affiliations:** 1 Department of Rehabilitation, Sint Maartenskliniek, P.O. Box 9011, 6500 GM Nijmegen, The Netherlands; 2 Center of Excellence for Rehabilitation Medicine, UMC Utrecht Brain Center, University Medical Center Utrecht, and De Hoogstraat Rehabilitation, Utrecht, The Netherlands; 3 Department of Rehabilitation Medicine, Center for Rehabilitation, University Medical Center Groningen, University of Groningen, Groningen, The Netherlands; 4 Department of Rehabilitation Medicine, Research School CAPHRI, Maastricht University, Maastricht, The Netherlands; 5 Adelante Centre of Expertise in Rehabilitation and Audiology, Hoensbroek, The Netherlands; 6 Rijndam Rehabilitation, Rotterdam, The Netherlands

**Keywords:** Spinal cord injury, Self-care, Self-management, Self-efficacy, Rehabilitation, Complications, Quality of life, Qualitative research

## Abstract

**Background:**

People with a recent spinal cord injury (SCI) often follow intensive rehabilitation. Learning appropriate self-care, deal with their impairments and prevent secondary health conditions (SHCs), is highly important during rehabilitation. To date it is not clear how self-care skills are taught to people with SCI. The objective of this study was to understand how people with SCI experienced the learning of appropriate self-care skills during inpatient rehabilitation, including the role of the rehabilitation team.

**Methods:**

Individual semi-structured interviews were conducted with 15 people with SCI, recently discharged from initial inpatient rehabilitation. Interviews were audio-taped, transcribed and analyzed thematically.

**Results:**

Two main themes and seven sub-themes were identified. Participants stated that the contribution of the rehabilitation team to learning self-care, including prevention of SHCs, was mostly made by *optimizing opportunities to learn through experience.* For preventing SHCs, education *and lessons learned from the professionals* during therapy and the formal educational program, was experienced as especially important. Further, *the motivational attitude* of the professionals which participants found stimulating and was based on respect, combined with their *positive contribution as one team,* were seen as essentials elements for learning appropriate self-care. However participants did not recognize the contribution of the nursing staff as part of their rehabilitation, although it was seen as very important. An important aspect of the participants’ own contribution was *challenging oneself to learn self-care*. This was done in different ways by the participants. Further, their own *mental adjustment* was considered important in the learning process. The *gaining of confidence* was by most participants seen as personal characteristic, although they also recognized the importance of the team effort and the experiences they underwent.

**Conclusions:**

Learning appropriate self-care was mostly done through experience, by challenging themselves, and making use of the opportunities given by the members of the rehabilitation team. The same strategies used by the rehabilitation team to teach people with SCI to perform appropriate self-care, were also helpful for the participants to gain confidence. Explicit attention for self-care training as an important goal in SCI rehabilitation may strengthen the nursing staff’s role and stimulate interdisciplinary working.

**Supplementary Information:**

The online version contains supplementary material available at 10.1186/s40359-021-00659-7.

## Background

Spinal cord injury (SCI) is a highly disabling condition [1, 2]. Besides the motor, sensory, and autonomic impairments, people with SCI are also at significant risk for secondary health conditions (SHCs), such as urinary tract infections, musculoskeletal and neuropathic pain, pressure injuries, edema and spasticity [3]. The primary impairments and the SHCs can limit people with SCI from participating in activities of daily life and reduce their quality of life [1, 4, 5]. To deal with their impairments and prevent SHCs, appropriate self-care is highly important for people with SCI, whether or not they are assisted by formal or informal caregivers.

Self-care can be defined as activities undertaken by individuals to promote their health, prevent disease, manage illness, and restore health as much as possible [6]. In research literature, self-care is often part of the broader term self-management, whereby self-management is defined as the individual’s ability to manage the symptoms, treatment, physical and social consequences, and lifestyle changes in accordance with living with a chronic disease (Chronic Care Model) [7]. In the current study we focused in particular on learning appropriate self-care, meaning how to deal with their impairments and how to prevent SHCs. Self-care by people with a disability not only depends on knowledge but also on skills and confidence in managing their condition, which is also referred to as self-efficacy [7]. Learning about self-care skills and preventing SHCs starts early in rehabilitation and reach a maximum level of independence before hospital discharge [8]. In the Netherlands this takes on average three months [9]. Both the increase of knowledge and the learning new or adapted skills are seen as an important part of the intensive SCI rehabilitation program [10, 11]. When people with SCI, learn early in their initial rehabilitation (first inpatient rehabilitation period after they sustained SCI) how to take care for themselves, this will prevent future SHCs, and maintains optimal health and well-being [11]. This learning is a complex process that requires involvement of the entire rehabilitation team [12, 13].

To date, there is not much academic attention on the process of learning self-care skills during initial SCI rehabilitation. Most studies on self-care among people with SCI focus on the chronic phase, or on a specific aspect of self-care, such as skincare [6, 14]. A study of people with a stroke or a SCI found that the experience gained from doing an activity, to become familiar with the impairments of the body, was a prerequisite for recapturing self-care during their initial rehabilitation period [15]. Members of the rehabilitation team play an important role in creating the context that contributes to learning self-care, by giving participants more time to catch up and become acquainted with their new situation, enabling patients to see possibilities, and create an expectation of being able to do things on their own [16]. In two reviews of qualitative studies, on the experience of rehabilitation following SCI, no studies were identified which had as their primary objective learning to perform self-care skills [17, 18]. Nevertheless, certain themes related to learning self-care were identified in these reviews. These themes included working together as a team; patients with the professionals. Participants described the importance of a supportive and encouraging team for successful rehabilitation [17]. Another theme was the importance of specific qualities of the team members, participants stated the importance of team members treating their patients as unique persons rather than rehabilitation clients [17]. Other themes were the team members’ vision about future life possibilities, building self-confidence (i.e. self-efficacy), and motivation were mentioned as themes [17, 18]. Increased self-efficacy during rehabilitation was described in multiple studies, enabling the participants to try new activities or to return to activities they performed prior to the onset of SCI [17]. Rehabilitation also played an important role in enabling the participants to be mentally and emotionally stronger which in turn helped to increase motivation [17]. A caring relationship with the members of the rehabilitation team helped and was described as a process in which participants started as passive recipients of information and gradually developed into active agents who had the opportunity to find out for themselves what they needed [19]. Interestingly, in a recent systematic review and meta-synthesis on self-care, no study was included that had, as their primary objective, learning the performance of appropriate self-care skills [8]. Most of the included studies focused on specific SHCs and were of community dwelling people with SCI. That meta-synthesis provided evidence of the self-care behaviors performed by people with SCI to prevent, control, and manage the physical, emotional, and social effects of their condition. Another study also noted that developing self-care skills is not always given sufficient priority by people with SCI and team members, because of a primary focus on the physical aspects of rehabilitation, insurance reimbursement policies, and cultural values associated with family caregiving [20].

To give sufficient priority to the self-care during the rehabilitation of people with SCI, it is necessary to know how people with SCI experience their role in learning self-care skills. Therefore the objective of this study was to understand how people with SCI experienced the learning of self-care skills during initial inpatient rehabilitation, including the role of the rehabilitation team in this learning process.

## Methods

### Study design

This study used a descriptive qualitative design. Data were collected using face-to-face semi-structured interviews. This approach was employed as there is a paucity of research on how people with SCI experience the process of learning self-care during their rehabilitation. The qualitative descriptive approach is considered a good starting point for topics about which little is known [21]. The study adhered to COnsolidated criteria for REporting Qualitative research Checklist [22].

### Participants

The present study is part of a large cohort study in the Netherlands (SELF-SCI), in which the development of self-management skills and self-efficacy after SCI is being investigated with quantitative and qualitative methods [23]. The SELF-SCI study has previously been described [23]. The inclusion for SELF-SCI in short; participants were admitted for their initial rehabilitation after they sustained a SCI, were > 18 years of age, had no severe cognitive impairment or severe mental health disorders, had no limited life expectancy, for instance due to cancer related SCI, were expected to be admitted for at least 4 weeks and possessed sufficient knowledge of the Dutch language to complete the questionnaires. For this particular qualitative part of the study, all participants of the SELF-SCI study discharged between February and May 2017, were asked to participate. Based on the hypotheses that self-efficacy is conditional for performing self-care, participants were divided into three groups. As part of the quantitative study, all participants completed a disability-management self-efficacy scale, the University of Washington Self-efficacy Scale 6 item version (UW-SES-6) [24, 25]. The scores on this questionnaire were divided into low (score 6–15), average (16–21) and high (22–30) based on all scores of the participants in the SELF-SCI study. We aimed to get an equal number of participants in each of the three groups, to get variation in self-efficacy that could be related to the experience of learning self-care. After analyzing the data of the first 13 participants, it turned out that the participants were not equally divided into the three groups; seven had a high score, three a middle score and three had a low score on the UW-SES-6. To investigate if including more people with low scores would give additional information, two more participants with low scores on the UW-SES-6 were invited in November and December 2017. These two interviews did not generate new themes or expand the understanding of how people with SCI experienced the learning of self-care skills during initial inpatient rehabilitation, so saturation was assumed.

### Procedures

Participants in the SELF-SCI study were informed about the purpose of this qualitative part of the study and could indicate in their discharge questionnaire whether they were willing to participate. Data collection took place in a single face-to-face semi-structured interview between two and five weeks after discharge from inpatient rehabilitation. The interview took place at the participant’s home or a rehabilitation facility, depending on the participants’ preference. In a few cases a partner was present during the interview. All interviews were digitally recorded and transcribed verbatim for data analysis. The Ethics Committee of the University Medical Centre Utrecht declared that this protocol did not need formal ethical approval under the Dutch law regulating medical research on human beings (reference number: 15-449/C). Next, in accordance with the local requirements, the Medical Ethics Committees of all participating rehabilitation centers approved this protocol. The study was carried out according to the code of conduct formulated by the Helsinki code, such that all participants gave written informed consent before entering the study.

### Interview

The interviews lasted between 45–70 min and were conducted by the first author, a trained male psychologist with 15 years of experience in SCI rehabilitation, assisted by two female trained master students. The participants had no professional relationship with the interviewer. The interview guide was developed based on scientific knowledge, by the investigating team and consisted of semi-structured open-ended questions. The interview guide was piloted and refined. Prompts were used during the interviews to get as concrete information as possible or to explore issues in greater depth and to verify the interviewer’s understanding of the information being collected.

The complete list of questions and prompts is included in Additional file 1: Appendix I. There were three major questions: how participants learned to take care of their body; how they learned to prevented SHCs; and how they gained confidence during their inpatient rehabilitation. For each of these aspects the participants were asked to describe what they had learned and how the members of the rehabilitation team contributed to this learning process.

### Data analysis

Data analysis was conducted in an iterative manner using inductive thematic analysis [21]. The first three transcripts were independently coded in full by two master students, giving attention to all data. The results were discussed with the first author until consensus was reached about the wording and meaning of the codes. After this open coding, axial coding followed in which already existing codes were refined, differentiated and linked with each other. The last step was selective coding, in which the core themes were identified, eighter other themes were linked together, or themes that needed more explanation were developed and refined. These results were discussed with the last author (MP) until consensus on the content and wording of the themes was reached. After this last adjustment, the coding framework was applied to all transcripts. MAXQDA 18.2 (VERBI Software, 2017) was used to organize and analyze the data.

## Results

### Participants characteristics

A description of the participants can be found in Table 1.

**Table 1:** Participant characteristics

Partici-pant	UW-SES-6	Age	Higher education^a^	Having a partner	SCI level start (end)^b^	AIS start (end)^b^	Days inpatient rehabilitation
A	29 (high)	55–60	No	Yes	T4 (L1)	D	91
B	26 (high)	60–65	Yes	Yes	C4 (C3)	D	91
C	25 (high)	55–60	No	Yes	T4	D	92
D	24 (high)	55–60	No	Yes	T10	D	44
E	24 (high)	50–55	No	Yes	C7	C (D)	113
F	24 (high)	40–45	Yes	No	C3	D	39
G	23 (high)	70–75	Yes	Yes	C4 (C5)	D	37
H	19 (middle)	60–65	Yes	Yes	T9	D	77
I	18 (middle)	65–70	Yes	Yes	T11	D	57
J	16 (middle)	30–35	Yes	Yes	T1	D	56
K	15 (low)	75–80	No	No	T12	C (D)	80
L	14 (low)	55–60	Yes	No	C5 (C6)	D	64
M	14 (low)	65–70	No	Yes	C3	C	203
N	11 (low)	65–70	No	Yes	T11 (L1)	C	85
O	11 (low)	55–60	Yes	Yes	C5 (C6)	A	250

UW-SES-6, University of Washington Self-efficacy Scale six item version; SCI, spinal cord injury; AIS, American spinal cord injury association Impairment Scale

^a^Higher education = bachelor degree or higher

^b^The end of inpatient rehabilitation is only given if there is a difference

The participants of the SELF-SCI study were on average admitted to one of the rehabilitation centers five weeks after the onset of their injury. Table 1 shows that almost all participants had an incomplete SCI, this is in accordance with the Dutch population of people with SCI admitted for rehabilitation. This explains why most participants stated that they did not need any assistance with their self-care at the end of inpatient rehabilitation or just needed a helping hand from their informal caregivers, mostly their partner. Three participants needed help from formal caregivers at the time of the interview, one because of the adaptations to their house was not ready, the other two because of insufficient function in their hands and arms.

### Themes

Participants reported that learning to perform self-care required both the contribution of the rehabilitation team as well as their own effort, and these two aspects seems to influence each other. These two components formed the basis of the two themes which were further divided into sub-themes. The findings are presented in Table 2.

**Table 2:** Structure of the core themes and sub-themes

**1 Contribution of the rehabilitation team**
Optimizing opportunities to learn through experience
Motivational attitude
Lessons learned from the professionals
Positive contribution as one team
**2 Participants’ own contribution**
Challenging oneself to learn self-care
Mental adjustment
Gaining confidence

## Contribution of the rehabilitation team

The rehabilitation program is an ongoing learning process involving many disciplines and activities. Therefore, for some participants, it was not always clear which activities could be labeled as learning self-care skills, which skills were taught by the members of the rehabilitation team and which they learned or adapted themselves. Nevertheless, most participants could distinguish between what they were taught by the members of the rehabilitation team and their own efforts to learn self-care. In the next part, the contribution of the rehabilitation team is described, and then the participants’ own contribution.

### Optimizing opportunities to learn through experience

Participants stated that members of the rehabilitation team purposefully created opportunities for them to learn appropriate self-care skills. Multiple strategies were mentioned by the participants, all with the over-arching goal themed as “optimizing opportunities to learn through experience”.

Quote participant J: (about how professionals of the rehabilitation centre advised her to take care of herself)

“By the physiotherapist, who gradually tried to improve the functioning of my arms and legs, step by step. And to get your strength back. In addition, the occupational therapist. They taught me to do everything on my own. They helped me with cooking, getting dressed and that sort of thing. And the nursing staff of course sometimes said: you can do that better. The doctors told about medication. So, it is the whole team who actually help you to reach your aims.

In case of the nursing staff, participants described learning self-care as a gradual process, completely integrated in their everyday routine. The nurses took over the complete care in the beginning and showed the participants what to do by describing the care they were providing, and why they did it in the particular way. Depending on the participants’ capabilities, they were expected to do more and more of the activity independently over time. Most participants mentioned this strategy as being very smooth, almost natural. Some experienced this approach as a (positive) pressure.

Quote participant G: (about the nurse’s role in teaching self-care) “For instance when you have to dress yourself, you ask a nurse; how can this be done? And she says; I will help you less and less.”

The occupational therapists used the strategy of optimizing opportunities to learn through experience to compensate for lost functions in day-to-day situations. The participants were told to perform the affected skills in a modified way or were told what aides could be used to compensate for lost functions, then this was put into practice. During the training, tips and tricks were added to get the best functional outcome.

Quote participant E: “They [occupational therapists] came and stood next to me, especially in the beginning. Then I had to do it [morning routine of washing and clothing] by myself, and they watched what went wrong. Then they intervened and told me how it could be done better, and what was easier.”

With respect to the physiotherapists, optimizing opportunities to learn through experience was seen, by most participants, as teaching step-by-step. Within this approach, first information was given about the goal and how it could be achieved, then the first small step was practiced before going to the next. Along the way old and new instructions were given, until the activity was learned.

Quote participant J: “Through the physiotherapist, who in stages, tried to improve all the arm functions and the leg functions. And to get the strength back.”

Quote participant A: (During walking practice) (imitate voice of physiotherapist) “What did we say: get your back straight and …. (in own voice of participant) Yes, I know it (laughing), Don’t take big steps”.

A specific aspect of optimizing opportunities to learn through experience, was applied by the participants who would not be able to carry out the care themselves. They had to learn how to instruct their (in)formal caregivers. This was mostly taught through experiencing the care in the rehabilitation center during the normal routine, especially by the nursing staff. While undergoing these daily routines the participants were taught how to instruct their future caregivers.

Quote participant O: “..and they [the team members] have always made clear: you are in control. That’s the way they bring you up. YOU are the specialist and actually it’s for you to teach the home carers. You must say precisely what you want; even though that’s sometimes annoying. You don’t want to have to explain it 100,000 times. But if you don’t say it; and if, for example, the catheter is lying under your leg, then you know for sure that you’ll be on your back for two weeks. So you’ve just got to notice it yourself.”

### Motivational attitude

Participants described multiple approaches applied by members of the rehabilitation team that were experienced as helpful for learning self-care skills. One often mentioned approach, used by different disciplines, was motivation of the participants. According to the participants, motivation had two aspects: stimulating them to take actions that they did not think were possible, and second showing conviction that the participant could master the task. This motivational strategy was used in different situations. For instance, by challenging the participants. This sometimes confrontational approach helped the participants to realize they could do more than they imagined.

Quote participant C: “At a certain moment they [nursing staff] challenge you. You did it last week, you did it yesterday also, …. so now you can do it yourself. And you will!”

On the other hand, team members were also protecting the participants against their desire to do too much themselves.

Quote participant I: “And then the nurses point out: Take care! You are asking too much from yourself. Call one of us to help you.”

Members of the rehabilitation team were described by many participants as resolute in their approach, which was experienced by most of them as stimulating their learning self-care skills.

Quote participant N: “And you’re inclined to say: today is an off-day, we shan’t do much. The physiotherapist knows how to handle that. (Laughing) They keep you to it …. and that’s good”.

Some members of the rehabilitation team were described as tough (sometimes too tough) and confrontational, while others were described as having a soft touch or being encouraging. One participant described how these different approaches, applied by different physiotherapists, worked for her.

Quote participant G: “The soft and the hard complement each other. He [male physiotherapist] was the unspoken force and they [two female physiotherapists] gave encouragement: you can do it, you’ve done it so far, come on”.

In addition, members of the rehabilitation team were described as being practical and not too cautious or afraid that something might go wrong.

Quote participant A: (About the nursing staff) “And they [nurses] weren’t afraid and not like ‘ieehh’ ..… No they just did it. Like myself, they have the attitude just do it, and if you can’t, then not and we’ll see it from there”.

Several participants stated that the normalizing attitude of the members of the rehabilitation team (e.g. nursing with regard to incontinence) helped them to overcome shame, gain confidence and therefore better be able to learn self-care.

Quote participant L: “In particular with bowels and urine. If that goes badly, you’re ashamed of yourself. They [nurses] say: you can’t do anything about it, we’re here to help you. They put you at ease and you needn’t be embarrassed”.

Some participants described a special mutual bond that they had with one or more team members. Those team members made them feel special. In general, most participants described the relationship with the various team members as respectful, and based on mutual respect. Because of this respect participants were able to receive emotional support from different team members.

Quote participant L: (about treatment from psychologist) “It is indeed important. Because I told my story to family and friends. But it’s good also to tell it to someone with whom you have totally different connection. …. (further on) That gives you a bit of peace actually, it’s the most important”.

### Lessons learned from the professionals

The participants found information on self-care, provided by the members of the rehabilitation team during the rehabilitation treatments and during the formal education meetings, very important. The information provided was helpful in understanding the importance of self-care and especially in preventing SHCs. Further, the information provided helped in understanding how to handle the possible consequences of SCI. This information could only be given by the team members because they had the specific knowledge about SCI and possible complications. The team members provided this information, and understood the participants needs well. A specific part of education that was mentioned by the participants was learning to recognize the changed signs and signals from the body.

Quote participant C: “Everything … so much in my body feels different from before. And if I go to the toilet, that too feels different. I’ve got to learn it again, to recognise it again”.

### Positive contribution as one team

When the participants were asked about the relative contribution of the different disciplines, with respect to learning bodily care after SCI, most participants answered by first describing what the occupational therapist or the physiotherapist did for them during the rehabilitation period. As if rehabilitation is the work of only the therapists. Most participants did not mention the nursing staff spontaneously. Nevertheless, when asked directly about the role of the nurses, many participants stated that the nursing staff was (one of) the most important disciplines in teaching them self-care activities, and also in preventing SHCs. Besides the nursing staff, occupational therapy and physiotherapy, all other disciplines from the multidisciplinary team were mentioned as contributing to self-care, although these were less pronounced.

Although the contribution of the different disciplines could be identified it was, for participants, not always easy to tell which discipline in particular helped them to learn self-care skills or to gaining confidence. Often this was not attributed to one discipline but seen as a team effort with all team members working together.

Quote participant B: “An effort of everyone [in the rehabilitation team], whereby you start doing things again as soon as possible - and dare to do and are not afraid of …. That applies also for the peer supporters. Actually to everybody, they all had a positive contribution”.

The participants perceived the attitude of members of the rehabilitation team, as described previously (1.3), to be part of the culture on the ward and therefore as a team effort. This included the respectful attitude, but also the motivational, as well as the implicit expectation of the team that participants would develop some sort of own initiative.

## Participants’ own contribution

### Challenging oneself to learn self-care

Many participants stated that learning appropriate self-care was very important to them. An important way to do this was by challenging themselves. This was done in various ways. For some it was by determining for themselves what they wanted to accomplish, both in rehabilitation as a whole as well as in activities they did during the day. To achieve the goals they set for themselves, participants needed to be persistent and inventive in finding solutions for activities they could not do. Further it was found helpful by the participants to see this process of achieving their goals as a contest.

Quote participant F: (about making a sandwich) “I wanted .…. to do everything myself, so I refused help. Thinking: if it takes me half an hour, then it takes me half an hour”.

Quote participant A: (about doing self-care himself) Yes. That was one of my aims, you know. To do everything myself as soon as possible. Even transferring myself, out of bed, into the wheelchair, from the bed to the walker.

Quote participant M: “Of course, you’ve got to do everything yourself, also go out on your own. So: as soon as I could manage the wheelchair, I went to the village, and to the beach, and that kind of thing”.

Some participants stated that they thought that other patients on the ward needed the helping hand of the nurses more than they did, and for that reason they did not ask for help.

Quote participant D: “Yes: they [nurses] help you and dress you ..… they are so ….. they have also got all those people with a complete spinal cord injury, those boys who need much more care. So you try to do as much as possible yourself, in order to spare them [the nurses], so I don’t keep on ringing the bell”.

For some participants, another way of challenging themselves was to search for information concerning their condition or SHCs on the internet. Also, some took the initiative to communicate with the therapists about adaptations, aides or a specific therapy which they felt could be useful to them at that time. Such challenging of oneself was time-consuming, but most of the participants perceived this strategy as a way to maximize the outcome of the rehabilitation treatment.

Challenging oneself was sometimes elicited by having to wait for help. In particular help from the nursing staff, but also from some occupational therapists, who were so busy that they were unable to respond to every request immediately. This seemed to be not a strategy used intentionally by the members of the rehabilitation team or the participants. Nevertheless, while waiting, a lot of the participants tried to do the activity themselves and found out ways to accomplish the task that they were asking help for.

Quote participant F: “And then I went to the toilet. I remember that I had just entered [name hospital], and I couldn’t wipe myself. And there I sat forgotten on the toilet. And then I thought: OK, I can’t do it well myself, but I’ll do it. Even if it takes me half an hour. I think that helped me a lot”.

### Mental adjustment

Participants described different ways of thinking, or a mindset, that helped them to get the best result from rehabilitation treatment, including self-care. One often mentioned mindset was thinking positively, instead of giving in to self-pity, or doing nothing which was not seen as directly helping.

Quote participant G: “Even if your body doesn’t work, you’ve to heal yourself mentally. You have to convince yourself you’re worthy, that you have something to give”.

Quote participant A: “But once again: I’ve got my illness and I’m going to solve it …. I’m not going to get depressed”.

Other participants stated that, as part of the mental adjustment, they had to accept that there were activities which they might not be able to do anymore. Not being able to do some activities as they did before their SCI, was confronting. At the same time, clarity about what they still would be able to do, and what not, seemed important to some participants, in order to accept their current situation. Most of these participants realized their condition could be worse. Some of them also mentioned the importance of resetting their priorities, for instance giving priority to family over work. Others talked about learning to enjoy the things they still could do.

### Gaining confidence

During the interviews, the participants were asked how they gained confidence in doing their self-care and in handling the consequences of SCI during their rehabilitation (self-efficacy). From the 15 participants, five scored low, at discharge, on a disability-management self-efficacy scale (see Table 1). Most of these low scorers actually described themselves during the interviews as having low confidence, but other low scorers said that they were fairly certain about themselves and the way they could handle the consequences of SCI.

Most participants stated that their confidence increased during the rehabilitation phase, although it fluctuated depending on their situation. For some participants, this increase depended, partly, on the natural recovery of functions after SCI. Therefore, physical setback would have damaged their confidence. Most participants experienced this increase in confidence as part of their personality, and not taught by professionals of the rehabilitation team. Nevertheless, many participants also mentioned that the experience they were given by the rehabilitation team, might have helped to gain confidence.

An increase in confidence was often associated with experiencing that they could indeed do a certain activity. The professionals of the rehabilitation team taught the participants through experiencing different activities (see 1.1), and this also increased the confidence of the participants.

Quote participant K: “It goes from wheelchair, to walker, to crutches, to a walking stick, and then to nothing. Every time your self-confidence increases”.

Furthermore, the participants mentioned that the professionals gave information about the expectation of recovery, or the influence of therapy on their functioning, which helped to increase confidence. Also, the second part of motivation (see motivational attitude), was ensuring the participants they could do a specific activity, and that this helped to increase their confidence. Lastly, some participants state that professionals reminded them about the progress they made.

Quote participant E: “They [the staff members] point out: … first you couldn’t do that, and now you can. That gives you a boost. (Partner) You could see that nicely when he sat on that special wheelchair, that you can click onto a handbike. Then he could go round the gym on his own. I have never seen him suddenly looking so enthusiast—‘I can do it again’. Wonderful to see”.

Some participants stated that confidence might have increased as a result of a combination of their own attitude, the contribution of others (team members, peers as well as family members) together with the experience of doing different activities and of returning to activities they did before SCI.

Quote participant E: (After talking about gaining confidence and the help from team members and partner) Interviewer: “What has given you back the most confidence? What has helped most?” Participant: “I can’t say what has helped me most. I think that it’s everything together. All the disciplines together. And yes—once again—my wife, she too was very important”.

Quote participant I: Participant: “I’ve got quite a lot of confidence in myself.” (Later in the interview) Interviewer: “how has [name of the rehabilitation center] contributed?” Participant: “By stimulating you in all sorts of ways. And creating the conditions under which you can make progress …. I mean: it’s there and it is offered to you. They see things sooner that you can”.

## Discussion

To understand how people with SCI experienced learning appropriate self-care skills during inpatient rehabilitation, including the role of the rehabilitation team, 15 people who had been recently discharged from clinical SCI rehabilitation were interviewed. The most important aspect about learning appropriate self-care, mentioned by the participants, was learning through experience. They did this by challenging themselves on the one hand, and being given opportunities by the team members. The application of optimizing opportunities to learn through experience varied between the disciplines as they had different roles within the team. Furthermore, the attitude of both the participants and the team members was found to be essential for this learning process. A motivating, stimulating, comforting and respectful attitude of the team members towards the participants was seen to be an important attitude of the professionals. This attitude was perceived to be embedded in the culture of the ward. The contribution towards the attitude of the participants themselves was for their own mindset to thinking positively and accept the current situation. Gaining confidence was, by most participants, seen as stemming from their own personality, nevertheless they also mentioned the contribution of the members of the rehabilitation team. For learning self-care, especially in preventing SHCs, information given during therapy and the formal educational program, was important for the participants. Much of the learning of self-care was considered a team effort, rather than the merit of just one discipline. Nevertheless, many participants distinguished between therapy and care, i.e. between physiotherapist/occupational therapists role and the nursing staff, with learning and rehabilitation is more attributed to the therapy role in first instance. However, when questioned most participants came to the conclusion that for learning self-care, all disciplines were important.

Learning through experience was found in previous research to be an important aspect of learning self-care [8]. Through giving the opportunity to get more experience, people with SCI are able to create new routines. Also the (motivational) attitude of both the professionals and participants was found in previous study to be of vital essence to learning self-care [8, 17, 18]. Aside from the motivation from the professionals, the mutual respect creates the base for learning [8, 17, 18]. In rehabilitation, alongside learning self-care, is also leaning other skills and knowledge. Therefore, several principles of adult learning are important [26, 27]. First of all, adults who are learning should be able to experience the skills to be learnt, bearing in mind their experience from their previous life. Secondly, adult learners like people with SCI in rehabilitation, learn best when convinced of the need to learn new or adjusted skills. Teachers should tap into the intrinsic motivation of the adult learner. This can be done by giving information on the goal and how to apply the new or adjusted skills on multiple occasions [26, 27]. In the current study all these aspects were described by one or more participants. Rehabilitation is a dynamic process in which many aspects including self-care are interwoven [13]. When teaching appropriate self-care, professionals use (formal) education, experiencing and gradually letting the participant do it themselves. In this way the three important learning styles of (adult) learners were applied; visual, auditory and kinesthetic [26].

To date there are, to our knowledge, no qualitative studies on learning appropriate self-care of people with SCI during inpatient rehabilitation. In two reviews of qualitative studies on the experience of rehabilitation following SCI many important aspects of rehabilitation in general were found, that also applied to learning appropriate self-care skills [17, 18]. One was about the role of the participants in thinking positively and accepting [17]. With respect to the role of the professionals, motivating the participants was found to be one of the main aspects, also the support given by the rehabilitation team, and working together as a team (members of the rehabilitation team with patients), were found important aspects of rehabilitation [17]. In other studies, a respectful professional caring relationship was found to be the foundation for learning in rehabilitation [19, 28]. In the present study, the participants stated that all these aspect also played an important role in the specific learning of appropriate self-care skills during SCI rehabilitation.

For the participants, it is not always clear when professionals are teaching self-care or other skills. Most participants seem to associate the rehabilitation process of learning new skills, including self-care, with the occupational therapists and physiotherapists, rather than with the nursing staff. Former literature has described how participants attribute the key role in SCI rehabilitation to the physiotherapist and the complementary role of the occupational therapist [29]. The distinction between the allied health professions and nursing staff has been found before, although this was from the professionals' point of view [30]. As a result of this segregation, nurses and allied health professionals were frequently seen as not working effectively towards the common goals; that is the goals of the people with SCI [30]. Making self-care training (e.g. dressing or washing oneself) an important and explicit goal in SCI rehabilitation might help to give the nursing staff a more ‘therapeutic’ status and at the same time intensify interdisciplinary working in the team.

During the interviews, there was special attention to the role of gaining confidence, that is increasing self-efficacy. Most participants stated that their confidence increased during the whole of rehabilitation, although there were fluctuations mostly due to physical setbacks. A qualitative study of people with stroke and SCI midway their initial inpatient rehabilitation found that most participants found themselves uncertain about learning self-care [15]. In contrast, participants in the current study experienced that they were quite certain about their self-care and about handling the consequence of SCI. Presumably, the second half of the rehabilitation process is needed to give participants the assurance they can handle their self-care.

Gaining confidence during rehabilitation is important because of the strong association between high self-efficacy with lower depression and anxiety, a higher quality of life and higher levels of participation [25, 31, 32]. Participants experience gaining confidence mainly as their own accomplishment belonging to their personality, and implicit to them, though they mentioned how different members of the rehabilitation team helped them to gain confidence. The team member did this by using the four sources of self-efficacy, formulated by Bandura; performance accomplishments, vicarious experience, verbal persuasion, and physiological feedback [33]. Although gaining confidence is important for rehabilitation outcomes, for participants gaining confidence is not experienced as an explicit goal during therapy or rehabilitation as a whole, which may have fed the idea that gaining confidence was their own accomplishment.

During the interviews, the participants with low scores on a disability-management self-efficacy scale did not all report themselves as being insecure in self-care or in handling the consequence of SCI. This could be explained by the fact that in a questionnaire specific behavior is being examined. Furthermore, people might be less inclined to admit that they were insecure during an interview than during (an anonymous) questionnaire. Most participants who had doubts about their confidence were not able to carry out the care themselves (at the time of the interview). This required a different sort of self-care learning, for which they had to develop skills to instruct their formal caregivers. These participants did not describe substantially different teaching methods by the team members. Although they were taught some different skills, the used strategies where basically the same. The fact that most of the participants who described themselves as uncertain during the interview also needed help from formal caregivers, might be a coincidence. In literature, there is no correlation found between self-efficacy and level or completeness of SCI [25, 34].

### Limitations

During the inclusion period of this study, there were more participants with a high score on the disability-management self-efficacy scale than those with a middle or low score. This might be because people with low self-efficacy scores were less willing to be interviewed which might have caused bias in the answers. However, including two more participants with low scores after the initial inclusion period, did not bring up new themes or expand the understanding how people with SCI experienced the learning of self-care skills during initial inpatient rehabilitation, and therefore we consider this might not have a big influence on the results.

Furthermore, the participants were recruited from different rehabilitation centers. This resulted in small numbers of participants per center. These small numbers made it hard to establish well-grounded differences between the centers. Nevertheless, substantial differences between the rehabilitation teams were not found. An advantage of this study is that the results did not come from only one center and are therefore more likely to be applicable to initial SCI rehabilitation in general.

Lastly most participants in this study were diagnosed with AIS D or C. Although this is the general picture in Dutch SCI rehabilitation it might have an influence on the results. As commented previously no substantial differences were found between participants that could or could not do the care themselves in how they were taught self-care skills.

## Conclusion

The most important aspect mentioned by the participants when learning appropriate self-care was learning through experience. They did this by challenging themselves and being given opportunities by the rehabilitation team. Those opportunities given by the professionals to experience self-care skills, were a very important aspect of gaining confidence.

Many participants distinguished between therapy and care, i.e. between physiotherapists / occupational therapists and the nursing staff. However learning appropriate self-care skills requires both roles; learning in a systematic way how to take care of a body with limitations, how to prevent SHCs and how to manage all consequences of SCI. Making self-care training as an important and explicit goal in SCI rehabilitation may help to give the nursing staff a greater ‘therapeutic’ status and at the same time stimulate interdisciplinary working in the team.

## Supplementary Information

**Additional file 1.** The complete list of questions and prompts used for the semi-structured interviews with people with SCI, regarding their experience of learning appropriate self-care skills during inpatient rehabilitation.

## Data Availability

The datasets used and/or analysed during the current study are available from the corresponding author on reasonable request. VERBI Software. (2017). MAXQDA 2018.2 [computer software]. Berlin, Germany: VERBI Software. Available from https://www.maxqda.com.

## Abbreviations

SHCs: Secondary health conditions
SCI: Spinal cord injury
UW-SES-6: University of Washington Self-efficacy Scale 6 item version
