# Supplementary material for: Learning self-care skills after spinal cord injury: a qualitative study
Source: BMC Psychol. 2021 Oct 9;9:155. doi: 10.1186/s40359-021-00659-7 (PMC8501583; doi:10.1186/s40359-021-00659-7)
Supplement: Supplementary file 1 — Additional file 1. The complete list of questions and prompts used for the semi-structured interviews with people with SCI, regarding their experience of learning appropriate self-care skills during inpatient rehabilitation. [file 40359_2021_659_MOESM1_ESM.docx]

**Additional files**

**File Name: Additional file 1**

**Title: Appendix 1**

**Description: Topic list used for the interviews**

Topic list

How are you today? What makes it good/bad?

1. How do you take care of your body?

Prompts:

- What do you do yourself, and what is done by others?
- How was this accomplished by [Name rehab center] during the rehabilitation?
- Which disciplines had contributed?
- How did these disciplines contribute?
- What has been helpful for you, in learning to take care of your body?
- What have you experienced as a barrier learning how to take care of your body?
- How did the spinal cord injury peer supporters contribute to learning how to take care of your body?
- How did your family/friends play a role in this process?
- What did [Name rehab center] teach your family/friends regarding your needed care?
- To what extent do you have the confidence that you can take care of your own body?
- Has the approach to treatment changed during the rehabilitation process?
- Have you missed anything in this process? What could be improved?

2. A spinal cord injury may lead to several health related problems, such as pressure injuries, pain, urinary tract infections or spasm. Do you recognize any of these problems yourself? Or do you have any other health related problems? What have you learned in the rehabilitation center to prevent these problems or to handle them if they occur?

Prompts:

- How was this accomplished by [Name rehab center] during the rehabilitation?
- Which disciplines had contributed to that?
- How did these disciplines contribute?
- How did the spinal cord peer supporters play a role in this?
- How did your friends/family play a part in this?
- How susceptible do you think you are for these health problems?
- Have you missed anything in this process? What could be improved?

3. People with a spinal cord injury may have different levels of confidence in how to live their life. How do you perceive the confidence you have in handling the consequences of the spinal cord injury?

Prompts:

- How was this accomplished by [Name rehab center] during the rehabilitation?
- Which disciplines contributed to that?
- How did these disciplines contribute?
- How did the spinal cord peer supporters play a role in this?
- How did your friends/family play a part in this?
- What did [Name rehab center] teach your family/friends with regard to your self-management?
- What has been helpful in handling the situation?
- What was a barrier to handling the situation?
- Has the approach to treatment changed during the rehabilitation process?
- Have you missed anything in this process? What could be improved?
